# Common mental disorders and subjective well-being: Emotional training among medical students based on positive psychology

**DOI:** 10.1371/journal.pone.0211926

**Published:** 2019-02-07

**Authors:** Leonardo Machado, Irismar Reis de Oliveira, Antonio Peregrino, Amaury Cantilino

**Affiliations:** 1 Postgraduate program in neuropsychiatry and behavioral sciences, Department of Neuropsychiatry, Federal University of Pernambuco, Recife, Brazil; 2 Department of Neurosciences and Mental Health, Federal University of Bahia, Salvador, Brazil; 3 Faculty of Medical Sciences, University of Pernambuco, Recife, Brazil; St. Paul's Hospital Millenium Medical College, ETHIOPIA

## Abstract

**Introduction:**

The prevalence of common mental disorders among medical students is globally high. However, medical students tend to seek less professional help to treat their mental health issues. Hence, ways have been devised to reduce emotional stress in this population.

**Objective:**

The current study uses positive psychology techniques to increase subjective well-being (SWB) in order to reduce symptons of common mental disorders (CMD) in medical students (MS).

**Methods:**

The study comprised two groups: intervention group (n = 37) and control group (n = 32). Throughout seven weeks, the intervention group had meetings focused on emotions, mental health of medical students, gratitude, appreciation, optimism, resilience, qualities and virtues. The control group attended conventional medical psychology classes (psychosomatic aspects in clinical illness, for example).

**Results:**

The intervention group presented average increase by 2.85 points in the positive emotions scale; average increase by 2.53 points in the satisfaction-with-life scale; and average decrease by 1.79 points in the SRQ-20 scale, when it was compared to the control group. The intervention effect size was moderate.

**Conclusion:**

Use of techniques to increase SWB may reduce CMD in MS, even if these techniques do not diminish negative emotions.

## Introduction

### Subjective well-being

In the field of positive psychology, well-being is divided into eudaimonic well-being (EWB) and subjective well-being (SWB). EWB, which is also called psychological well-being, is linked to the realization of intimate potential, and consists of some parameters such as positive relationships and self-acceptance. SWB, which is also called hedonic well-being, is connected to the experience of satisfaction, and has a cognitive component (satisfaction with life) and an affective component (positive emotions). There are validated scales to measure these different types of well-being which include published neuroimaging studies to access their respective neural correlates. It is important to emphasize that happiness is sometimes seen as a synonym for SWB. However, the tendency is to consider it as the main affective component of SWB. In this work, this trend was followed because it is the most current [1–5]

### Common mental disorders

The term common mental disorder is widely used to encompass depression, anxiety, and somatization disorders. They can be said to be the modern equivalent of neurotic disorders [6]. These disorders lead in terms of prevalence and clinical morbidity in the field of psychiatry [7].

### Mental health of medical students

The presence of anxiety disorders, depression, and burnout syndrome in medical students is generally high [8–10]. Among the risk factors there are variables associated with the personality of the student [11], the challenges inherent in the process of becoming a doctor [12], and the excessive stress of graduation [10,11]. In general, the mental health of medical students worsens throughout the course of medical training as they tend to develop dangerous coping strategies, such as alcohol abuse, in addition to seeking less medical help for their psychological problems, even though they are immersed in healthcare settings [13]. Mental illness and high psychological stress are associated with difficulties in the doctor-patient relationship [14,15] and academic performance [11,14]. Therefore, efforts have been undertaken to manage stress factors in medical students [16].

### Positive psychology

Positive Psychology (PP) is a new field of psychology that seeks to understand positive emotions, psychological potential, and healthy human/social/institutional functioning, as well as to apply this knowledge to help people and institutions. It has particular focus on preventive measures and the promotion of mental health [2,17,18].

Although PP effectively began to develop and have an organized approach since the publication of Seligman and Csikszentmihaly's work, which serves as its basis [19], the constructs of PP started being studied decades earlier. Even the name PP cannot be credited to the authors, since Maslow, one of the leading names in humanistic psychology [20], was the one who used the expression for the first time in a chapter of his book "Motivation and Personality", published in 1954 [2,21,22].

Regardless, PP is nowadays immersed in the main field of study of topics such as positive emotions, human strengths/potentials and virtues, hope, gratitude, appreciation, optimism, among others [2,5,23–29].

### Interventions based on positive psychology

From the beginning, PP theorists were concerned not only with developing psychotherapeutic techniques, but also scientifically measuring and evaluating its efficacy [18]. At present, there are descriptions of group, individual, institutional, and internet based intervention [28,30–35].

In general, PP intervention is technically similar to Cognitive-Behavioral Therapy (CBT) [36,37]. However, in the same manner as the research, intervention not only focuses on people with psychiatric diagnoses or psychological problems, but rather, anyone [23,34,37,38]. Therefore, it can be said that intervention focuses on the promotion of well-being and the development of human potential, regardless of the presence or absence of any psychological stress or psychiatric disorder. The goal is not to reduce symptoms, but to stimulate and enhance healthy characteristics.

Within scientific literature, the authors have found two meta-analyses evaluating the efficacy of these therapeutic proposals [39,40]. The oldest, from 2009, [39] involved 51 studies and found a significant increase in well-being (primary outcome) and a decrease in depressive symptoms. However, an important limitation of this study is that the authors included intervention that was not originally derived from positive psychology, such as *mindfulness*, making the method very heterogeneous and perhaps overestimating its effectiveness.

In fact, given the ample focus of PP, it is natural for it to have many points in common with other proposals, such as *mindfulness* [41–43], and *coaching* [34]. However, it is important to verify whether the therapeutic proposals of PP alone are effective. This is precisely what the second reported meta-analysis did. By analyzing 39 studies, it was found that the PP intervention was effective in increasing subjective and psychological well-being, and in decreasing depressive symptoms. The effect size of this second meta-analysis was about 0.3 points smaller than the effect size of the meta-analysis performed by Sin & Lyubomirsky [40].

### Positive psychology and medical education

Despite medical students being a risk group for mental illness, and positive psychology having developed well over the last two decades, few initiatives based on positive psychology have emerged in order to strengthen protective factors for all medical students, regardless of the presence of mental illness [44]. It is believed that the application of various aspects of positive psychology in medical education can have a positive impact on professional development [14].

### Aim and hypothesis of this study

Through this perspective, the present research sought to create preventive emotional training for medical students based on positive psychology techniques, concepts of emotional neuroscience, and behavioral genetic insights.

The authors' hypothesis is that by introducing preventive emotional training based on positive psychology to medical schools' curricula, it is possible to increase SWB scores and decrease the scores that evaluate symptoms of common mental disorders in medical students.

## Methods

### Outline

This is an intervention study (longitudinal, prospective, and with psychotherapeutic intervention). Members of both groups (intervention and control) were chosen at convenience since the corresponding author was the professor of the classes that constituted the two groups, and one of the co-authors was the coordinator of the teaching module in which the intervention was introduced, thus permission for the experiment to be included in the medical curriculum was granted. The volunteers, the researcher, and the statistician were not unperceptive in relation to the groups.

### Data collection, groups, and ethical aspects

The data was collected through a self-administered questionnaire applied to sixth and seventh-semester medical students from a Brazilian university before and after seven weeks of intervention. The study comprised two groups, namely: intervention group (n = 37, students attending the seventh semester of medical school), and control group (n = 32, sixth-semester medical students). The number of students participating in the groups was equal to the number of students enrolled in the classes.

The intervention was employed during a seventh-semester course because one of the co-authors is its coordinator, thus authorizing the introduction of the experiment in the said course. The control group was composed of sixth-semester students since the corresponding author was the professor of the sixth-semester course. In the following semester, students in the control group also received the same emotional training.

It should be emphasized that three students from the intervention group and a student from the control group were excluded from the statistical analysis because they participated in less than 60% of the meetings. Therefore, in the statistical analysis, the intervention group had n = 34, and the control group had n = 31.

Anonymity was required in the questionnaire response. Before the volunteers could participate in the research, they were provided with oral and written explanations, and they signed a free and informed consent form in accordance to resolution 466/12. The volunteers did not receive any allowance, and all research expenses were paid for by the main researcher.

The two groups were informed of the research and signed a consent form; their anonymity was assured. The study was approved by the Research Ethics Committee of the Health Sciences Center at the Federal University of Pernambuco, authorized by its Medical School Board of Directors, and registered in the Brazilian Registry of Clinical Trials (ReBEC) (Registry Number: RBR-6zmz3c).

### Tools

A self-applicable questionnaire was created based on the following components: socio-demographic data; extracurricular activity data; Satisfaction with Life Scale (SWLS); Positive and Negative Affects Schedule (PANAS); and, Self-reporting questionnaire (SRQ-20). An evaluation with questions concerning the quality of the classes/meetings, and also the professor's commitment to the process, was conducted at the end of the seventh week, and is included in the study.

The SWLS [45, 46] is a scale that is used worldwide to measure the cognitive component of subjective well-being; it was translated and adapted to countless countries in many languages (12, 41), including Brazilian Portuguese, for more than a single demographic context [47–49]. The SWB cognitive component is related to the judgment an individual makes about his own life as a whole. It is a brief unifactorial tool comprising 5 Likert-type questions, whose responses range from 1 ("I strongly disagree") to 7 ("I fully agree"). Therefore, the minimum score is 5 (the lowest satisfaction) and the maximum is 35 (the highest satisfaction) (12, 41, 45). The scale has good internal consistency (Cronbach's alpha = 0.87) and can be used in any age group, from adolescence and beyond (12, 41, 45).

The PANAS is a self-report scale composed of 10 adjectives that represent positive affective states assessing positive affects, as well as of 10 items assessing negative affects (a score between 10 and 50 is generated for each affect type) [50]. The responses to each item are based on a five-point Likert scale, wherein 1 means "not at all" and 5 means "extremely". It can be applied to any adult population, and it is one of the scales that evaluates different affect types, which is most commonly used internationally (12, 41). The scale has already been translated and validated for the Brazilian population, being used even by university students. It possesses high internal consistency for both subscales (Cronbach's alpha of 0.88 for the positive emotions subscale, and 0.87 for the negative emotions subscale) [51].

The SRQ [52] was proposed by the World Health Organization (WHO) for mental health care strategies, mainly for primary health care (19). The 20-item version (SRQ-20) was designed to screen mood, anxiety, and somatization disorders, which are collectively known as common (or minor) mental disorders. These conditions account for 90% of the total morbidity caused by psychiatric diseases. Nowadays, the SRQ-20 is appropriately used in many contexts other than primary health care. The best cutoff point is 7/8 for both women and men [53]. The present study used 8 as the cutoff point. The scale has already been translated and validated in Brazil [54]; there are even more recent validations [55] and studies confirming its good performance in the screening of common mental disorders [56, 57]. It has very good internal consistency (Cronbach's alpha of 0.86), and an accuracy of 91% [53].

In addition, two questions concerning the quality of the meetings and the professors' commitment to the classes were added to the final questionnaire. Since there was no blinding in the study, and the professor/psychiatrist who performed the intervention was the same for both groups, and there was an objective search for any data that could reveal a possible position taken by the researcher that could strongly bias the research.

Basically, they were asked to choose a number from 1 to 5, in which 1 would represent "completely disagree", and 5 “completely agree", regarding the following two sentences:

- “I believe that the teacher was committed to my class."
- "I consider the quality of the classes, the debates, and the topics addressed as being good."

### Description of the emotional training based on positive psychology

For seven weeks, the control group received conventional medical psychology classes. Topics that were explored included empathy; death and dying in medical practice; aggressiveness in medical practice; oncology and terminal illness; ICU, nephrology, and psychiatric inter-consultations; psychosomatic aspects of gastroenterology; endocrinology, dermatology, and psychiatric inter-consultation. Some classes were taught by the professor, and some topics were addressed through seminars and videos that were presented by the students under the guidance of the professor.

In parallel, and also for seven weeks, the intervention group had its classes from the module of medical psychology transformed into emotional training based on the discoveries of the science of well-being and the techniques of positive psychology in the following order: two meetings with theoretical discussions about the neuroscience of negative and positive emotions, behavioral and optimistic genetics, the mental health of medical students and physicians; three encounters addressing positive emotions (gratitude, appreciation, and optimism) and resilience; two meetings about qualities and virtues. In addition to experiences during the meetings, scientific articles and technical proposals of positive psychology were provided between encounters. The meetings took place on a weekly basis and lasted for two hours. Below, there is a brief discussion about each of the intervention groups' meetings. The number of seven weeks/seven meetings was determined by the duration of the academic semester.

At the first meeting, the research was explained, the students signed the free and informed consent form, and filled out the questionnaire. Subsequently, it was followed by a discussion that derived from the question: What is the reason for an intervention when someone is "well"?

From there, data was collected regarding the mental health of physicians and medical students, which indicated that they were a risk group for psychiatric illnesses. Two scientific articles on the subject were handed out for reading at home, and debates occurred at the beginning of the second meeting. Each half of the class received a different article [11,58].

Then the question arose: would it be possible to discuss mental health prevention? From the contributions of the students, the so-called "emotional brain" was discussed [59–66]: basic history of neuroscience; historical and neuroanatomical bases of behavior and emotions, negative emotions, psychiatric disorders, positive emotions, and well-being; the basic mechanisms of emotional regulation; and, finally, mental health prevention studies.

Turning to the subject of the mental health of doctors and prevention in psychiatry, the class ended with the question: Is it desirable and is it possible for doctors to undergo intervention which aims at increasing their positive emotions and potential during training?

In the second meeting, the topics of the previous meeting were briefly summarized, and the two articles that were handed out the previous week were discussed. The end of the debate revolved around the last question that was raised at the end of the first meeting. Thus, positive psychology (history, concepts, and meta-analyzes on the effectiveness of intervention) was introduced [18,19,40] and news quickly spread about the so-called positive psychiatry [67].

The topic of personality was then emphasized, mainly through discussion of Cloninger’s model of temperament and character [68,69], and the most current data regarding gene-environment interaction in the formation of personality and mental illness [70].

The first two meetings were more theoretical. Their aim was to provide a knowledge base for the medical students that participated in the intervention process on current topics regarding genetics, neuroscience, psychiatry, and psychology.

An article on the genetics of optimism was made available for study between the weeks that had debates and the third meeting [71]. The third meeting began by briefly summarizing the main points of the second meeting and debating the scientific article that was made available.

From this point on, the process of leaving theoretical discussions and entering practical psychotherapeutic propositions began, thus posing the following questions: Why experience techniques? Why not just study and think about the subject? As a point of observation regarding the difference between cognitive knowledge and emotional knowledge, the example of empathy was used, which can be divided into affective and cognitive empathy [72]. The discovery and the implications of mirror neurons was also discussed [73,74].

**Fig 1:**
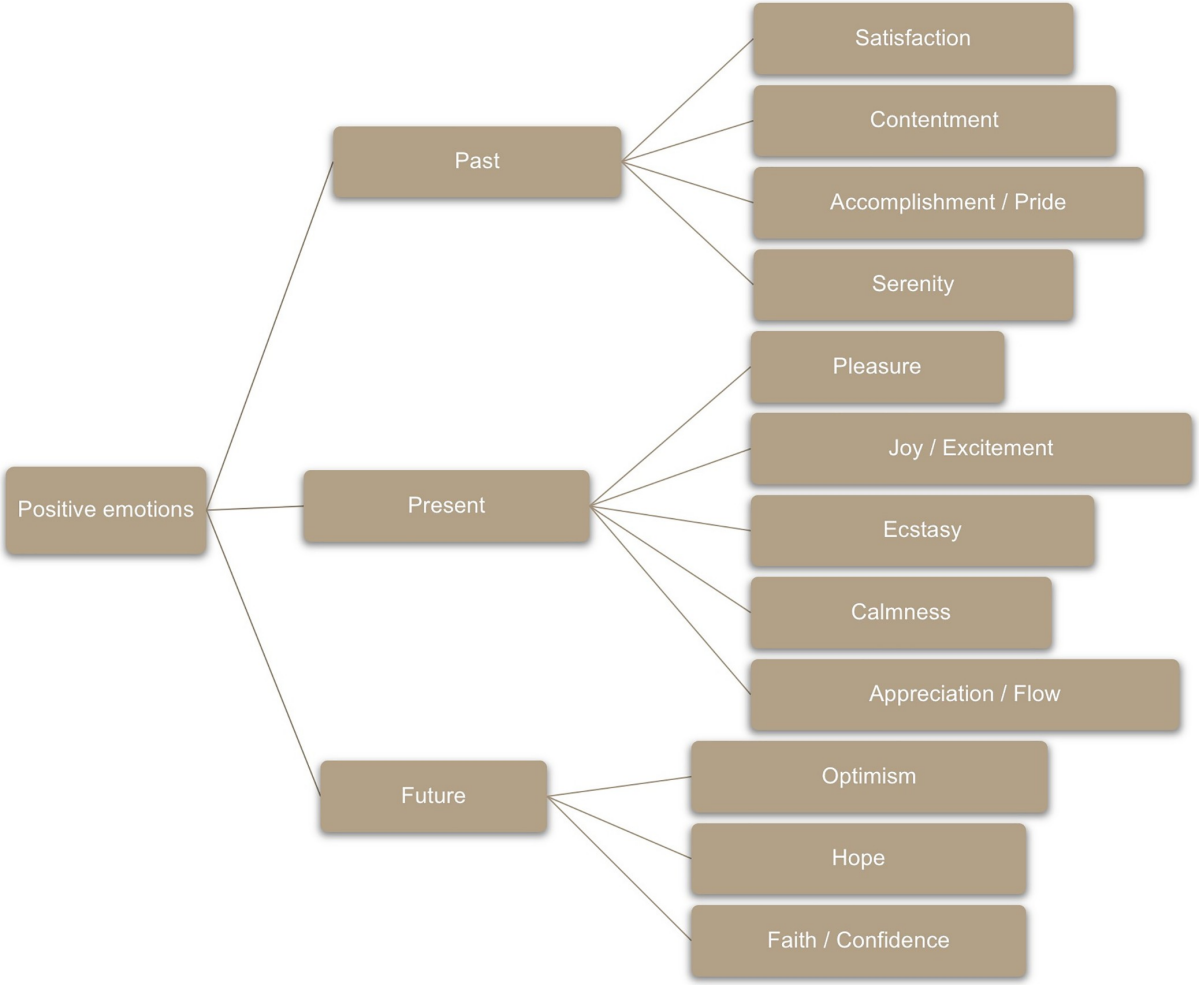
Didactic division of positive emotions according to positive psychology.

Then the work with positive emotions began. It was based on the didactic division proposed by positive psychology [36], as shown in Fig 1.

As a way of increasing positive emotions related to the past, there was focus on gratitude [23,36,75,76]. Themes such as selflessness and selfishness (or egotism) surfaced, being widely debated [23,77]. In the end, the techniques known as the counting of "blessings" and a gratitude visit were recommended [18,23,32,35,36].

The fourth meeting began with activity sharing and the resumption of Fig 1. The idea was to work the emotions related to the present and future. For this reason, a discussion began on the verification of human suffering through various sources of knowledge: Heidegger, Kierkegaard, Schopenhauer, Buddha, Krishna, and Jesus. It was debated that everyone can observe the experience of suffering that is inherent to human condition, although their future visions are divergent in terms of what can be done in relation to the suffering [78,79]. Based on this, several aspects of optimism, seen as a positive emotion, with attachments to the future, were emphasized; also, there was a debate on the advantages, disadvantages, and common aspects of optimism and pessimism (in their most varied degrees) [34,36,80–82].

Subsequently, the focus turned to the positive emotions linked to the present, especially appreciation (savoring) and *flow* [23,30,42,83,84]. Some of these aspects are similar to *mindfulness* [41], however *mindfulness* is not exactly considered positive psychology [40]. In fact, *mindfulness* is part of the so-called third wave of CBT [85]. Since positive psychology itself has technical similarities with CBT [36], it would be natural for it to have similarities with *mindfulness*. However, for the emotional training proposed here, specific concepts or techniques of *mindfulness* were not used.

Regardless, during the meeting, songs were used for appreciation. Initially, they had no aesthetic explanations, and in a second moment, technical considerations were presented regarding the musical score and/or the song interpretation. In the end, the following techniques were proposed, as well as "screen time decrease" [23], and it was also suggested that students seek to enjoy and savor activities of personal interest according to some principles of savoring [86].

In the fifth meeting, the topic of optimism was resumed and the "Seligman optimism exercise" technique was proposed. This technique uses the cognitive model to identify thoughts and emotions [36]. From this, the ability to postpone gratification [87, 88] and resilience [23,34,89–91] began to be discussed. One important point of emphasis from this class was the analysis of the search for existential meaning as one of the mechanisms involved in resilience [23,92–94]. Subsequently, the focus shifted to personal qualities as being another mechanism that promotes resilience. Furthermore, a therapeutic activity was proposed which would be performed between weekly meetings: talking to patients who demonstrated more resilient attitudes towards their illness, especially chronic diseases.

The last two meetings were devoted to personal strengths, virtues, qualities, and potentials. The author produced videos that emphasized, according to the categorization proposed by positive psychology, the following groups of virtues: wisdom, courage, humanity, and justice [17,23,28,29,36]. In addition, excerpts from Viktor Frankl's story were debated in order to emphasize transcendence [94]. In these meetings, the class was divided into small groups in order for them to create discussion among themselves, and subsequently share their thoughts with the whole class. As a psychotherapeutic technique, the Values in Action Inventory of Strengths (VIA-IS) [36] tool was used to carry out the activities "Identifying personal strengths" and "Identifying forces in a family member/person of personal reference".

At the end of the seventh meeting, the students filled out the questionnaire again, but this time with questions related to professor assessment and the quality of the meetings, as well as an open-ended question about what each person felt about having experienced emotional training based on positive psychology. Subsequently, the participating students were given a text with the summary of activities and techniques along with the encouragement that they continue practicing the techniques over the coming weeks after the end of the emotional training. Table 1 summarizes the structure of the proposed intervention.

**Table 1:** Structure of emotional training based on positive psychology.

Meeting	Goal	Content	Techniques	Guiding questions
1^st^ meeting	Introducing the intervention Providing scientific background to the intervention	Presenting the intervention Mental health of medical students and physicians Neuroscience of negative and positive emotions Emotional regulation Prevention in Psychiatry	Theoretical presentation using audiovisual resources Discussion in the classroom Handling over two scientific articles: one about happiness in psychiatrists and another one about mental health in medical students	Why making an intervention when one is well? Is it possible talking about prevention in mental health?
2^nd^ meeting	Providing scientific background to the intervention	Positive Psychology Cloninger’s Personality Model Genetics of behavior and emotions	Debate about the articles handled over at the 1^st^ meeting Theoretical presentation using audiovisual resources Discussion in the classroom Handling over a scientific article about the genetics of optimism	How do environment, genetics, and personality influence the emotional states?
3^rd^ meeting	Encouraging positive emotions	Affective and cognitive empathy Positive emotions linked to the past according to Positive Psychology: gratitude	Debate about the articles handled over at the 2^nd^ meeting Counting the blessings Gratitude visit	Why applying positive psychology techniques and not just studying and thinking about welfare? Is it possible lessening the impact of negative memories from the past?
4^th^ meeting	Encouraging positive emotions	Positive emotions linked to the present according to the Positive Psychology: appreciation Positive emotions linked to the future according to Positive Psychology: optimism	Debate about human suffering Optimism vs. Pessimism vs. Cognitive distortions Music appreciation Decreasing screen time In pursuit of appreciation	Are optimism and pessimism direct consequences of the suffering one undergoes or does not undergo? How to enjoy pleasant events better?
5^th^ meeting	Thinking about steps to facilitate a resilient attitude	Resilience Meaning of life	Seligman's optimism exercise Learning from resilient people	How can positive emotions and meaning of life contribute to resilience?
6^th^ meeting	Encouraging strengths, virtues and qualities	Strengths, virtues and qualities according to Positive Psychology	Videos showing examples of people experiencing the virtues VIAS questionnaire used to identify personal strengths	How can personal virtues contribute to well-being, resilience, and professional activity?
7^th^ meeting	Encouraging strengths, virtues and qualities	Strengths, virtues and qualities according to Positive Psychology Summary of the intervention and encouragement for the participants to keep on practicing the techniques	Videos showing examples of people experiencing the virtues VIAS questionnaire used to identify strengths in family members and/or reference people Text containing the summary of the herein approached topics and techniques	How can personal virtues contribute to well-being, resilience, and professional activity? Can the understanding about the qualities of others help improving relationships?

### Statistical analysis

The sociodemographic characteristics and the data regarding extracurricular activities were initially compared. Pearson's Chi-square test was used to compare proportions, whereas the Student's t-test was used to compare the means. The Student's t-test was used to assess the effect of the intervention on related samples. Distribution was normal in all scales according to the Shappiro-Wilk normality test, except for the SRQ-20 measurements. Two forms of analysis were conducted to assess the SRQ-20: the first compared the medians by using the non-parametric Wilcoxon test for related samples; the second categorized scores according to the likelihood of mental disorder using the McNemar Chi-square test for related samples.

For the analysis between groups, the difference between evaluations carried out before and after the intervention was calculated in relation to the PANAS, satisfaction with life, and SQR-20 scales. Subsequently, linear regression was performed considering the difference between the measurements taken before and after as a response variable, in which the estimated regression coefficient represents the mean difference between intervention and control groups. The model parameters for each of the scales were estimated independently. The effect size of the intervention was estimated through the Cohen test.

The Stata software, version 12.0, was the statistical software used in the analysis; the statistical significance was set at 5% (p <0.05).

## Results

The two groups were similar in the following variables: gender, marital status, children, moving to another city to attend medical school, people they live with, type of housing, receiving financial help from parents, feeling uncomfortable for receiving financial help, not undertaking paid work, carrying out leisure activities, carrying out artistic activities, participating in socializing events, performing physical activity, and BMI. There was a statistically significant difference between the average age of the groups (23.8 ± 4.1 in the intervention group; 21.8 ± 1.5 in the control group; p 0.013), and the percentage of students performing extracurricular activities during the time the study was conducted (100% in the intervention group; 83.9% in the control group; p 0.015). After seven weeks, the students were asked to give their opinion on the teacher's commitment towards them, and on the quality of the classes and/or intervention. There was no significant difference of opinion among students belonging to both groups (Table 2).

**Table 2:** Characteristics of the intervention and control groups.

Characteristics	Intervention (n = 34)	Control (n = 31)	p-value
**Gender:** female	17 (50,0%)	18 (58,1%)	0,515
**Age** (average ± sd)	23,8 ± 4,1	21,8 ± 1,5	**0,013**^**a**^
**Marital status:** Single	33 (97,1%)	30 (96,8%)	0,947
**Has children**	1 (3,3%)	1 (3,8%)	0,918
**Moved from another city to attend Medical School**	13 (38,2%)	12 (38,7%)	0,969
**With whom they live:** Parents and siblings	25 (73,5%)	23 (74,2%)	0,440
**Type of Housing:** Apartment/House	34 (100%)	31 (100%)	1,000
**Currently performs extracurricular activity**	34 (100%)	26 (83.9%)	**0,015**^**a**^
** On-call duties**			
1 per week	23 (71.9%)	10 (76.9%)	0,729
2 per week	9 (28.1%)	3 (23.1%)	
Extension Project	19 (55.9%)	20 (64.5%)	0,478
Research Project	13 (38.2%)	16 (51.6%)	0,279
**Performed extracurricular activity**	33 (97.1%)	30 (96.8%)	0,947
On-call duties	24 (70.6%)	20 (64.5%)	0,601
Extension Project	29 (85.3%)	29 (93.5%)	0,284
Research Project	21 (61.8%)	18 (58.1%)	0,761
**Employment is not related to medical course**	2 (6.1%)	1 (3.2%)	0,592
**Receives financial support from family/parents**	34 (100%)	30 (96.8%)	0,298
**The financial aid bothers**	19 (55.9%)	10 (33.3%)	0,089
**Performs leisure activity**	31 (91.2%)	30 (96.8%)	0,348
Daily	4 (12.9%)	4 (13.3%)	0,816
Weekly	6 (19.4%)	4 (13.3%)	
Monthly	21 (67.7%)	22 (73.4%)	
**Performs artistic activity**	9 (28.1%)	8 (26.7%)	0,898
**Usually becomes involved with events to socialize**	21 (61.8%)	22 (71.0%)	0,434
**Performs physical activity**	20 (58.8%)	20 (64.5%)	0,638
Less than 3 times/week	12 (60.0%)	11 (55.0%)	0,946
3 or more times/week	7 (35.0%)	8 (40.0%)	
Daily	1 (5.0%)	1 (5.0%)	
**BMI (kg/m**^**2**^**) (average ± sd)**	23,7 ± 3,6	22,7 ± 3,7	0,268
**On a scale of 0 to 5 points, how would you rate the teacher's commitment to the class?**			
4	2 (5.9%)	3 (11.5%)	0.294
5	32 (94.1%)	23 (88.5%)	
**On a scale of 0 to 5 points, how would you rate the quality of classes/meetings?**			
4	3 (8.8%)	4 (15.4%)	0.475
5	31 (91.2%)	22 (84.6%)	

^a^ Difference was statistically significant (p < 0.05)

The group that was subjected to the intervention showed significant increases in positive emotions and satisfaction with life when drawing a comparison with before and after the intervention. In addition, the intervention group showed a reduction in the number of students with scores higher than or equal to 8 in the SRQ-20 scale (the highest risk score for which there is presence of mental disorders). Before the intervention, 32.3% of the students showed risk scores that indicated common disorders; however, this proportion decreased to 23.5% after the intervention. These three parameters did not show significant differences in the control group when they were compared before and after the experiment (Table 3).

**Table 3:** Intra-group comparison of scales.

Scales	Intervention (n = 34)			Control (n = 31)		
	Before	After	p-value	Before	After	p-value
**Positive and negative affect schedule (PANAS)**						
Positive affect	32.8 ± 6.1	36.0 ± 5.4	**0.003**^**a**^	33.5 ± 4.8	33.9 ± 6.0	0.691
Negative affect	22.5 ± 8.8	20.9 ± 7.6	0.186	23.9 ± 7.5	22.1 ± 6.2	0.089
**Satisfaction with life scale (SWL)**						
Score	23.3 ± 6.9	25.4 ± 6.1	**0.009**^**a**^	23.4 ± 6.3	23.0 ± 7.0	0.425
**Self-reporting questionnaire (SRQ-20)**						
Score ^c^	6 (2; 9)	4.5 (3; 7)	0.084	6 (4; 11)	8 (5; 12)	**0.047**^**a**^
Possibility of having common mental disorders ^b^	11 (32.3%)	8 (23.5%)	**0.005**^**a**^	13 (41.9%)	17 (54.8%)	0.836

^a^ Statistically significant difference (p < 0.05)

^b^ Prevalence (%)–Mcnemar's test

^c^ Median (P_25_; P_75_)–Wilcoxon test

The intervention group showed an average increase of 2.85 and 2.53 points in the positive emotions and satisfaction-with-life scales respectively, as well as an average decrease of 1.79 points in the SRQ-20 scale, when compared to the control group. The effect size of the intervention was moderate (Table 4).

**Table 4:** Comparison of scales between the intervention and control groups.

Scales	Regression coefficient		p-value	Effect size^a^
	β	CI 95%		
**Positive and negative affect schedule (PANAS)**				
Positive affect	2.85	0.27 a 5.44	**0.031**	0.55
Negative affect	-0.28	-3.35 a 2.80	0.858	-
**Satisfaction with life scale (SWL)**				
Score	2.53	0.68 a 4.39	**0.008**	0.49
**Self-reporting questionnaire (SRQ-20)**				
Score	-1.79	-3.13 a -0.46	**0.009**	0.35

^a^ Cohen's test: moderate effect between 0.3 and 0.7

## Discussion

A previous study that was conducted by the corresponding author on the SWB of medical students from the same Brazilian public university found low levels of SWB and high levels of anxious concern in the referred population [95]. Thus, emotional training based on positive psychology was elaborated. The present work that evaluates the effectiveness of this intervention is within the UFPE medical program. This intervention can be considered preventive because there are several levels of prevention (primary, secondary, tertiary). Because we use the classroom (on the principle that positive psychology is called positive education), it was not possible to exclude students who were already very likely to have a psychiatric diagnosis. All the students needed to have the opportunity to receive the classes transformed into a training, since it was part of their academic curriculum. Thus, it is not possible to classify the intervention as a primary prevention. The focus was to increase protective factors such as life satisfaction and positive emotions (SWB), and to not directly decrease risk factors such as psychiatric symptoms. Thus, a seven-week program was developed, and it was incorporated into UFPE’s medical program curriculum. In the end, the training that is proposed and analyzed here was effective in increasing scores on the positive emotions and life satisfaction scales (which are used to measure SWB), and in reducing the SRQ-20 score (which is used as screening for common mental disorders). Interestingly, even though it was not effective in decreasing the negative emotions scale score, the intervention showed efficacy in decreasing the SRQ-20 scale score.

Thus, the current study has shown that using techniques to increase SWB throughout medical training helps to decrease psychiatric symptoms among medical students, although it does not diminish negative emotions. It may be a way of reducing common mental disorders within this population. The effectiveness of Positive Psychology-based interventions in increasing SWB and reducing psychiatric symptoms, mainly the depressive symptoms, was also demonstrated in other environments [18,33] and in at least two meta-analyses [39,40]. At least one intervention method based on Positive Psychology had already been proposed for the medical student population, which was essentially based on strengths and virtues; however, unlike the present study, the efficacy of such intervention was not subjected to statistical analysis [44].

In addition, recent systematic reviews have corroborated the efficacy of mental health intervention applied to medical students [16,96]. The herein conducted intervention was performed in the context of an undergraduate program, i.e., within the program curriculum. However, the best scenario to promote these interventions—whether within the program curriculum, as proposed in the current study, or outside the academic curriculum—is not clear, so far. Further investigation may expand this preliminary data, using larger samples, and searching for the best intervention format in order to promote mental health among medical students: within the curriculum (having the advantage of not increasing the extra workload of students, as well as involving all students, even those who do not initially believe that improving well-being and preventing illness is necessary), or as an extra-curricular activity (having the advantage of forming smaller groups, which tend to start the intervention well motivated). In addition, future investigation may help to identify students who could benefit the most from this type of program.

It is interesting to note that, consistent with what is seen in the medical student's mental health literature[13], the mental health of the control group appears to have worsened throughout the semester, since psychiatric symptoms have increased. This seems to be a favorable evidence for the creation of interventions aimed at improving the mental health of medical students.

It is important to explain that the students who, for some reason, sought the individual help of the researcher were attended in individual psychotherapy or in clinical psychiatry by psychiatric residents of UFPE. In fact, a program of psychiatric and psychotherapeutic assistance in partnership with UFPE's medical residency program in psychiatry was created [97].

The results from the current study also lead to considerations about the very nature of mental disorders. The fact that the negative affect scale scores did not decrease, but the SRQ-20 scores were reduced, allows one to believe that psychiatric symptoms, mainly psychiatric diagnoses, are more complex than the simple sum of negative emotions. Likewise, the so-called well-being does not seem to be just the absence of negative emotions, in the same manner that health is not simply the absence of disease.

On the other hand, the current study seems to corroborate one of the main Positive Psychology proposals, namely: the protective effect of positive emotions and well-being [36,82]. These aspects appear to have a buffering effect on negative emotions, as well as on the suffering associated with mental disorders and other clinical diseases. Therefore, working with these elements seem to present another window of opportunity to establish preventive efforts within psychiatry, not only for medical students, but for other populations as well.

In fact, it is possible to question whether the effects that were found in the present study will remain long term. Thus, further investigation is necessary. However, it is possible to considerer that, just as the effect of psychotropic drugs for the treatment of mental disorders depends on continuous use for a relatively long time, it is advisable, or at least desirable, that the participants continuously apply the techniques that were taught in order to prolong their effects. In fact, this happens with other psychotherapeutic techniques; hence, the students were instructed to keep practicing these techniques after the end of the intervention.

The present study possesses some limitations, which should be taken into consideration. The students in the intervention group were slightly older than those in the control group. Such age difference could lead to a more mature confrontation of adversities. The two groups were from different semesters in the medical program, a fact that may be associated with different specific stress sources. In addition, the study only involved MS. There was no masking of investigators and participants. Finally, the feeling of being taken care of and/or observed, as well as of having spent seven weeks without having to be concerned about being academically evaluated by the herein proposed program, may have made the intervention group feel better.

The fact that a single researcher taught the classes that were attended by the control group, and also conducted the meetings attended by the intervention group, could potentially be a bias. However, the current study has examined this possibility by questioning the participants on the professors' commitment to the groups, and on the quality of the classes and meetings. Both groups attributed high scores to the professor who conducted the study; there was no significant difference between the groups' opinions. Thus, what might have been bias seems to have become a positive aspect of the present study. If different professors had conducted the research in each group, the differences in the results could have been attributed to possible differences in the quality of the student-professor relationship, i.e., a possible bias that would be more difficult to overcome.

It is possible that the good quality of the professor-student relationship (deduced from the score that the students gave the professor) might have contributed to the positive results of the intervention. In fact, the good quality of human relations established between therapist/doctor and patient is an aspect that must always be taken into consideration, and used as an ally when dealing with psychiatric disorders and mental suffering.

Although mental illness conditions among medical students are known worldwide, preventive intervention proposals with desirable statistical accuracy remain scarce, especially in Latin America.

## Conclusions

The preventive emotional training that is based on the findings of well-being science and on Positive Psychology techniques, which was proposed over a seven-week span within the context of the medical program at a Brazilian university, has effectively increased life satisfaction and positive emotions, while decreasing psychiatric symptoms among medical students.

The data also indicates the possibility of expanding the preventive efforts of psychiatry based on the promotion of mental health resources. Further research could increase the medical students' sampling group, verify the long-term effects of intervention, assess whether intervention is more effective within or outside the program curriculum, and finally, extrapolate the findings to different populations.
